# Altered expression of striatin-4 is associated with poor prognosis in bladder transitional cell carcinoma

**DOI:** 10.3892/ol.2021.12592

**Published:** 2021-02-25

**Authors:** Yuhan Zhang, Xinquan Gu, Fuquan Jiang, Pinghui Sun, Xu Li

**Affiliations:** 1 Department of Urology, China-Japan Union Hospital of Jilin University, Changchun, Jilin 130033, P.R. China; 2 School of Public Health, Jilin University, Changchun, Jilin 130015, P.R. China

**Keywords:** striatin-4, bladder cancer, overall survival time, metastasis

## Abstract

Striatin-4 (STRN4 or Zinedin) is a scaffolding protein belonging to the mammalian STRN family of proteins and consists of multiple functional signaling domains. Due to its numerous signaling complexes, STRN4 has been reported to be involved in the tumorigenesis of various cancer types, including colon cancer, liver cancer and prostate cancer. However, few studies on STRN4 have been conducted in bladder cancer, and its prognostic role in bladder cancer remains unknown. The present study aimed to investigate the expression levels of STRN4 in bladder transitional cell carcinoma and evaluate the prognostic role of STRN4. STRN4 expression in clinical specimens was analyzed using immunohistochemistry and reverse transcription-quantitative PCR. It was demonstrated that STRN4 expression was significantly associated with clinical parameters such as tumor size, muscle invasion depth and pathological tumor grade. Abnormal STRN4 expression was typically associated with worse overall survival time and outcome when compared with the low STRN4 expression group. Using multivariate analysis, it was reported that STRN4 was an independent prognostic biomarker for survival time in bladder transitional cell carcinoma. Although the specific biological mechanisms of STRN4 in bladder cancer still remain to be elucidated, STRN4 expression could be a prognostic indicator in bladder cancer.

## Introduction

Bladder cancer has been categorized as the ninth most commonly diagnosed cancer worldwide in 2016 and ranked as the fourth in the USA in 2018 (1). Unlike other malignancies, the morbidity and mortality of bladder cancer remained stable from 1975 to 2014 despite oncology advances in novel medicine treatment or diagnosis over these years, even with small increases in morbidity and mortality rates during a specific time period (2). The 5-year survival rate of patients with bladder cancer was 73% between 1994 and 1998 and 76% between 2009 and 2013 worldwide (3). These data indicate that current treatment approaches targeting bladder cancer have not significantly improved when it comes to prognosis. Hence, novel therapeutic procedures are urgently needed.

Striatin-4 (STRN4), also known as Zinedin, is a scaffolding protein of the mammalian STRN family of proteins that do not exert any enzymatic functions but serve as regulatory cascade subunits for protein phosphatase 2A (PP2A) (4). The STRN family consists of three highly homologous proteins: STRN, S/G_2_ Nuclear autoantigen (SG2NA) and STRN4. These three proteins manifest in a conservative manner due to their presence both in fungi and higher eukaryotes (5). Numerous studies have reported that the STRN proteins are expressed in various organs such as the central nervous system (6), lung, kidney and testis (7). However, the exact expression pattern differs, which indicates that the STRN family of proteins possess abundant but differential functions at the organ level or cellular level (8). For example, STRN is found to be expressed both in the central and peripheral nervous system, but mainly in the striatum and motor neurons. In addition, STRN is involved in the control of motor neurons and dendrite growth. SG2NA is highly expressed in the cerebellum and cortex, but it is most highly expressed during the S and G_2_ phases, which indicates its extensive involvement in cell cycle control (4,9). Similar to its homologous proteins, STRN4 contains four conserved functional domains that allow STRN4 to interact with numerous signaling pathways, such as the estrogen receptor signaling (STRN4-ERα) and RhoA pathways (10). These complex structural domains define its extensive involvement in biological and pathological processes. Previous reports have demonstrated that STRN4 participates in cell cycle regulation, Golgi assembly, apoptosis, neuron development and tumorigenesis, especially concerning its role in vascular formation (8,11,12).

Abnormal STRN4 expression can promote cell cycle progression and assist cancer cells in gaining invasive properties by activating downstream transcription factors, including misshapen-like kinase 1, RhoA, TRAF2 and NCK-interacting protein kinase (belonging to germinal center kinases) and platelet-derived growth factor receptor-α (PDGFRA) (13). Reznickova *et al* (13) have revealed that STRN4 functions as a fusion protein during the activation process of PDGFRA and accelerates the development of chronic eosinophilic leukemia (11,13). In another study, STRN4 was reported to promote cell proliferation and inhibit apoptosis in the prostate under the regulation of leukemia/lymphoma-related factor (14). However, to the best of our knowledge, there are no studies investigating the expression pattern of STRN4 in bladder cancer and the association between and STRN4 expression and prognosis.

The present study investigated the prognostic role of STRN4 in bladder cancer and aimed to determine the association between STRN4 and recurrence.

## Materials and methods

### 

#### Fresh samples and tumor specimens of bladder cancer

Up to 28 fresh excised bladder transitional cancer tissues along with 28 normal bladder tissues (3–3.5 cm away from the tumor) were collected after surgical removal at the China-Japan Union Hospital of Jilin University (Changchun, China) between December 2015 and December 2017. None of these patients had received anticancer therapy (including chemotherapy and targeted medicine) before surgery. The samples were immediately stored in liquid nitrogen (−196°C) for further experiments. A total of 112 bladder transitional cancer specimens with different Tumor-Node-Metastasis (TNM) stages (15) were obtained from the Department of Tissue Bank, China-Japan Union Hospital of Jilin University. Informed consent was obtained from all patients and the study was approved by The Ethics Committee of China-Japan Union Hospital of Jilin University (Changchun, China).

#### RNA harvest and reverse transcription-quantitative PCR (RT-qPCR)

Total RNA was extracted from excised bladder cancer and normal tissues using TRIzol^®^ reagent (Invitrogen; Thermo Fisher Scientific, Inc.) and reverse transcribed into cDNA with SMART^®^ MMLV Reverse Transcriptase (cat. no. 639524; Takara Bio, Inc.). The mixture was heated at 70°C for 3 min, cooled down and subsequently centrifuged at 17,344 × g for 1 min at 4°C, incubated at 50°C for 60 min, and a final extension at 70°C for 15 min. RT-qPCR was performed to detect STRN4 expression using the 2^−∆∆Cq^ method (16) with the PowerUp™ SYBR™ Green Master Mix (cat. no. A25742; Applied Biosystems; Thermo Fisher Scientific, Inc.) according to the manufacturer's standard protocol. Primers were designed using the GPP Web Portal (https://portals.broadinstitute.org/gpp/public) and screened using the Blast function from both Primer Premier 6 (version 6.00; Premier Biosoft) and National Center for Biotechnology Information. STRN4 primers were purchased from Shanghai GenePharma Co., Ltd. The primer sequences were as follows: STRN4 Forward, 5′-GATCTCACCGTCACCAACGA-3′ and reverse, 5′-GGAACGAATGCCGTCGTAGT-3′. GAPDH was chosen as an internal control. qPCR was performed on the QuantStudio™ 5 Real-Time PCR system (Applied Biosystems; Thermo Fisher Scientific, Inc.). Initial denaturation at 95°C for 30 sec followed by 40 cycles of three-step PCR amplification comprising denaturation for 5 sec at 95°C, annealing for 34 sec at 60°C, and extension for 10 sec at 72°C.

#### Immunohistochemistry (IHC)

A total of 112 previously acquired formalin-fixed, paraffin-embedded bladder transitional cancer specimens along with 28 fresh samples and 28 matched adjacent non-cancerous tissues (ANCT) were cut into 4-µm thick slices and analyzed as previously described (17). Two pathologists who were blinded to the experimental data were invited to score each specimen's immunostaining degree using a light microscopy (Olympus Corporation; magnification, ×40). The images were captured using CellSens (version 2.3; Olympus Corporation). The immunostaining intensities were set as follows: 0 (No stain=blank), 1 (weak stain=light yellow), 2 (moderate stain=light brown) and 3 (strong stain=brown). In addition, the positive immunostaining proportions were categorized into four tiers: 0 (<10%), 1 (11–25%), 2 (26–50%) and 3 (>51%) (18). The intensity and proportion values were multiplied as the composite index. The marginal index (threshold) was set at 3. Specimens with composite index scores ≤3 were considered to have a low expression of STRN4. The STRN4 primary antibody (cat. no. ab230858; Abcam) titer was set at 1:100. Mouse IgG1 κ Isotype Control P3.6.2.8.1 (cat. no. 14-4714-82; eBioscience; Thermo Fisher Scientific, Inc.) was diluted at 1:100. IHC was performed under standard procedures according to the Abcam manufacturer's protocol.

#### Statistical analysis

Data are presented as the mean ± SD (unless otherwise shown) with each experiment performed in triplicate. Statistical analysis between two groups was evaluated using two-tailed unpaired Student's t-tests and Fisher's exact tests using GraphPad Prism 7 (GraphPad Software, Inc.). The association between STRN4 expression and clinicopathological parameters of bladder transitional carcinoma was analyzed using the χ^2^ test. The survival data of up to 165 patients were collected from the epidemiology and statistics database (jointly built with First hospital of Jilin University) from the School of Public Health, Jilin University. Survival curves were established using the Kaplan-Meier method and compared using log-rank tests with SPSS 26.0 (IBM Corp.) and multivariate analysis was conducted using a Cox regression model. P<0.05 was considered to indicate a statistically significant difference.

## Results

### 

#### STRN4 is overexpressed in bladder transitional carcinoma

**Figure 1.**
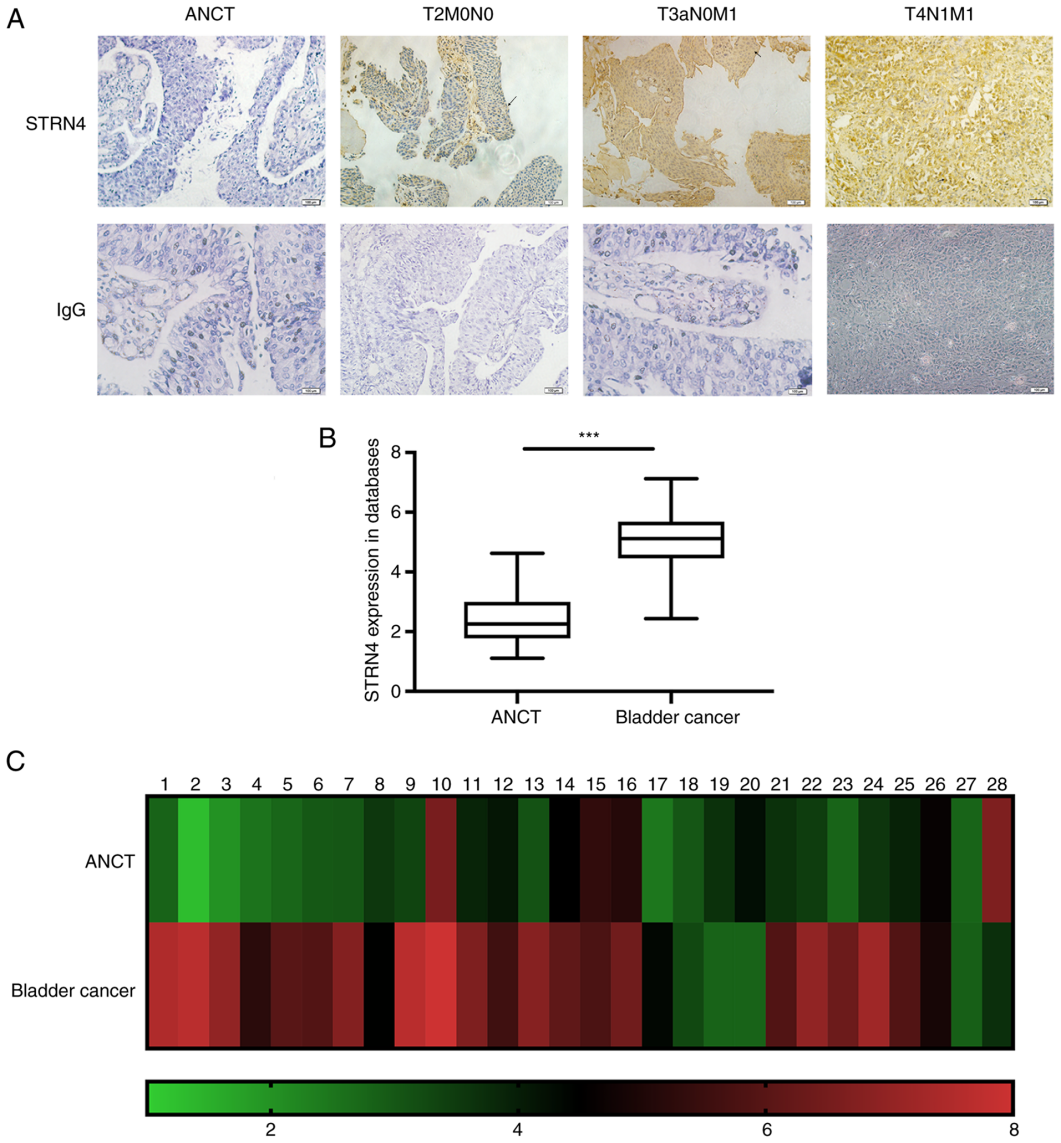
STRN4 expression in postoperative bladder transitional cancer samples. (A) STRN4 expression in the ANCT group and different TNM stage specimens. Magnification, ×40. (B) Relative STRN4 mRNA expression of patients with bladder carcinoma from the database. (C) Relative STRN4 mRNA expression of 28 fresh bladder cancer samples in heat map form. Green indicates low expression whilst red indicates high expression. ***P<0.001. STRN4, striatin-4; ANCT, adjacent non-cancerous tissues; TNM, Tumor-Node-Metastasis.

In order to determine STRN4 expression in bladder transitional cancer, IHC was performed on 28 pairs of fresh bladder tumor samples. As shown in Fig. 1A, the location and expression of STRN4 varied in different TNM stages. In relatively early TNM stages, STRN4 was mainly located at the cell membrane. Upon progression of TNM stage, STRN4 was located primarily in the cytoplasm and with increased expression. T2N0M0 indicates stage II in TNM stages. T3aN0M1 and T4N1M1 represents stage IV in bladder cancer.

Moreover, the present study analyzed STRN4 mRNA expression of patients with bladder carcinoma from the epidemiology and statistics database from the School of Public Health, Jilin University (19). STRN4 was significantly overexpressed in cancer samples (43 cases) compared with ANCT (23 cases) (P<0.001; Fig. 1B). Additionally, STRN4 mRNA expression in 28 paired samples was examined and presented as a heat map (Fig. 1C). RT-qPCR verified that STRN4 mRNA expression was significantly increased compared with the ANCT group by an average of 1.81-fold (maximum, 6.05-fold; P<0.001; data not shown). In summary, these results suggested that STRN4 is overexpressed in human bladder transitional cell carcinoma.

#### Aberrant STRN4 expression is associated with clinicopathological features

**Figure 2.**
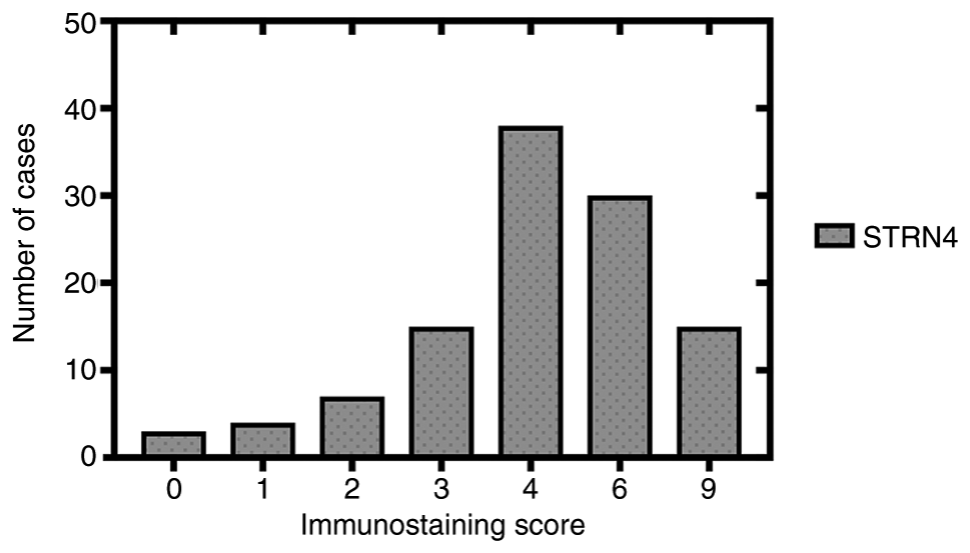
Immunostaining scores of STRN4 expression from 112 clinical bladder transitional cancer specimens in graphical form. Horizontal coordinates represent immunostaining scores (0, 1, 2, 3, 4, 6 and 9), which consists of different immunostaining intensities (0, 1, 2 and 3) multiplied by the immunostaining proportion (0, 1, 2 and 3). STRN4, striatin-4.

In order to investigate the association between STRN4 expression and specific clinical parameters, certain features such as age, sex, tumor size and muscle infiltration were chosen to measure the effects of STRN4 expression. As shown in Table I, STRN4 expression was significantly associated with tumor size (P=0.005564), muscle infiltration (P=0.000357), pathological tumor grade (P=0.001500) and microvascular invasion (P=0.034741). However, STRN4 expression was not statistically associated with age (P=0.542109), sex (P=0.240841) and lymph node involvement (P=0.232024). High STRN4 expression in patients with high grade bladder carcinoma was 84.2% (59/70) compared with 57.1% (24/42) in patients with low grade bladder carcinoma. STRN4 was detected in 77.5% (69/89) of cases with deep muscle infiltration (T2-T4) compared with superficial tumor invasion (Ta-T1), which was 39.1% (9/23). In patients with microvascular invasion, the detection rate of STRN4 was 81.5% (53/65) compared with 63.8% (30/47) in the low expression group. The immunostaining scores of 112 samples are listed in graphical form (Fig. 2). The number of specimens with different immunostaining scores are listed below: 3 (score 0), 4 (score 1), 7 (score 2), 15 (score 3), 38 (score 4), 30 (score 6), 15 (score 9). High STRN4 expression (score >3) was detected in 74.1% (83/112) of cases compared with cases with low expression (score ≤3), which was 25.9% (29/112).

#### High STRN4 expression in bladder transitional cancer is associated with adverse survival outcomes

**Figure 3.**
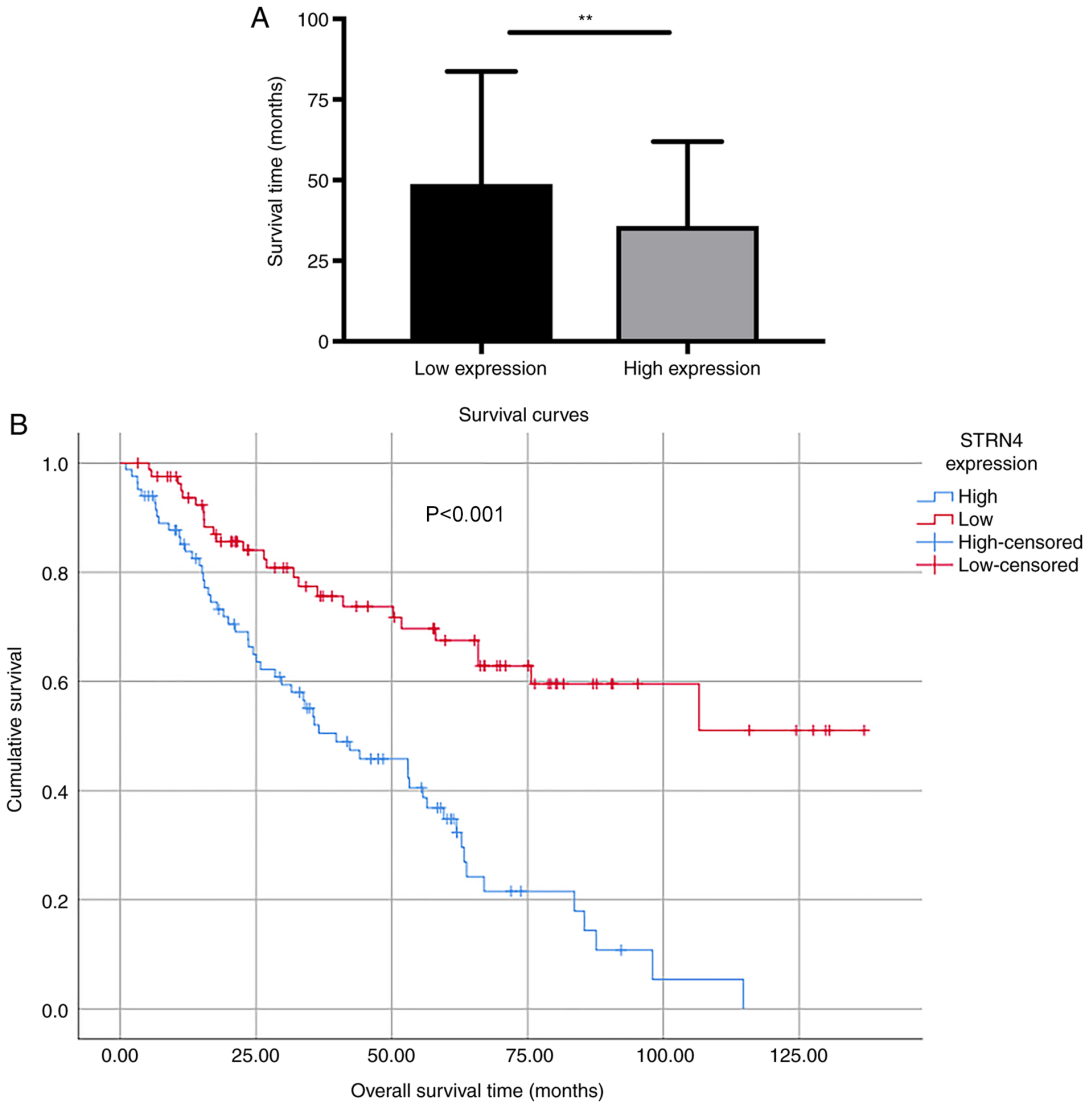
Survival analysis of STRN4 expression. (A) Different survival time of patients with low STRN4 expression and high STRN4 expression. (B) Kaplan-Meier analysis of overall survival time of patients with bladder transitional carcinoma according to STRN4 expression. **P<0.01. STRN4, striatin-4.

As shown in Fig. 3A, the mean survival time of patients with high STRN4 expression was 35.82±2.874 months (n=83), which was significantly lower compared with the low expression group (48.77±3.86 months; n=82; P=0.0077). Kaplan-Meier survival curves demonstrated that patients with high STRN4 expression ended up having a significantly shorter overall survival time compared with patients with lower STRN4 expression (P<0.001; Fig. 3B). Multivariate analysis was conducted to determine whether STRN4 expression could serve as a risk factor. STRN4 expression, along with other possible prognostic factors, were analyzed using Cox regression analysis. As shown in Table II, low pathological tumor grade could serve as a protective factor (coefficient=−0.951). The risk level of patients with low pathological tumor grades was 0.386 [hazard ratio (HR)=0.386] when compared with the high tumor grade group. The risk of patients with no relapse was 0.242 [hazard ratio (HR)=0.242] compared with patients with recurrence. STRN4 expression could serve as an independent factor for evaluating prognosis in patients with bladder transitional carcinoma (P=0.0018).

## Discussion

Bladder transitional cancer is known for its frequent recurrence after transurethral resection. The probability of 5-year recurrence for patients (mainly from UK, France, Spain and Netherlands in 2016) with European Organization for Research and Treatment of Cancer grades 1–4 is ~46%. Higher grade is accompanied by higher recurrence possibilities (20–22). Hence, identifying novel indicators of recurrence is particularly important for patients with bladder tumors.

Through proteomic analysis, researchers have noted that STRN4 is included in the polyprotein complex known as STRN-interacting phosphatase and kinase (STRIPAK) as one of its core components (8,12,23). STRIPAK consists of several crucial proteins such as PP2A, germinal center kinase III, cerebral cavernous malformation 3 and monopolar spindle one-binder family 3 (24–26). Accumulating evidence has indicated that STRN4 participates in the development of various cancer types and cardiac dysfunction via STRIPAK and STRIPAK-like complexes (27,28). Other reports have stated that the dysregulation of STRN4 expression exhibits potential for diagnosis and prognostic prediction (29–31). However, the prognostic role of STRN4 in bladder cancer is unclear and needs to be further determined.

The present study first examined STRN4 expression in postoperative samples. The results demonstrated that STRN4 was distinctly overexpressed in bladder transitional cancer samples when compared with ANCT. Meanwhile, STRN4 mRNA expression was analyzed using qPCR and compared with database results. The present study concluded that STRN4 mRNA expression was significantly higher compared with the ANCT group and the overexpression pattern was consistent with database results. These results indicated that STRN4 expression was upregulated in bladder transitional cancer.

To further elucidate the clinical relevance of STRN4, the association between STRN4 and several clinicopathological indexes were analyzed. STRN4 was significantly associated with tumor size, muscle infiltration, pathological grade and microvascular invasion. Muscle infiltration, pathological grade and microvascular invasion were listed as some of the most crucial factors for evaluating patient survival time and prognosis. The association between these variables and patient outcome is not strictly confined to bladder cancer, but also in other malignancies (32–34). Hence, STRN4 may serve a role as an independent prognostic factor. Furthermore, since the main metastasis approach for bladder transitional carcinoma is still muscle intrusion, these results suggested that STRN4 is important in tumor metastasis and recurrence, and may serve as an effective indicator for progression.

To determine the prognostic value of STRN4, survival analysis was performed to determine the effects of STRN4 on patient OS time. It was demonstrated that patients with high STRN4 expression had a lower OS time compared with the low expression group. Multivariate analysis indicated that STRN4 was an independent positive prognostic factor for OS. Taken together, the present results demonstrated that STRN4 may be a potential indicator for the prognosis of patients with bladder cancer and could be a new therapeutic target. To conclude, the present study indicated that STRN4 is an independent prognostic factor and potential therapeutic target for patients with bladder transitional carcinoma.

The present study is not without limitations. First, the analysis between STRN4 expression and the clinical parameters only consisted of 112 samples. Larger sample sizes are required to validate the results presented here. In addition, STRN4 mRNA expression analysis was performed using 43 cancer samples and only 23 ANCT samples. For prospective studies, equivalent samples are required to exclude interference from confounding and mismatching factors. Further studies are also required to determine the molecular mechanisms underlying the abnormal expression of STRN4 in the tumorigenesis of bladder transitional carcinoma.

## Figures and Tables

**Table I.** Correlation between STRN4 expression and clinicopathological parameters in patients with bladder transitional carcinoma.

		STRN4 expression		
			
Clinical pathological variable	Number of patients	Low, n (%)	High, n (%)	P-value
Total	112	29 (25.9)	83 (74.1)	
Age, years				0.542109
<65	35	12 (34.3)	23 (65.7)	
≥65	77	22 (28.6)	55 (71.4)	
Sex				0.240841
Male	81	36 (44.4)	45 (55.6)	
Female	31	10 (32.3)	21 (67.7)	
Tumor size, cm				0.005564
<3	82	31 (37.8)	51 (62.2)	
≥3	30	6 (20.0)	24 (80.0)	
Muscle infiltration				0.000357
Ta-I	23	14 (60.9)	9 (39.1)	
T2-T4	89	20 (22.5)	69 (77.5)	
Lymph node involvement				0.232024
No	100	46 (46.0)	54 (54.0)	
Yes	12	5 (41.7)	7 (58.3)	
Pathological tumor grade				0.001500
Low	42	18 (42.9)	24 (57.1)	
High	70	11 (15.8)	59 (84.2)	
Microvascular invasion				0.034741
No	47	17 (36.2)	30 (63.8)	
Yes	65	12 (18.5)	53 (81.5)	

STRN4, striatin-4; T, tumor.

**Table II.** Multivariate analysis of the association between clinical indexes and survival time of patients with bladder cancer.

							95.0% CI for HR	
							
Variable	Coefficient	Standard error	χ^2^	df	HR	P-value	Lower	Upper
Age, ≥65 vs. <65 years	0.022	0.010	4.633	1	0.0314	1.022	1.002	1.043
Sex, male vs. female	−0.193	0.294	0.432	1	0.5112	0.824	0.463	1.467
Muscle infiltration, T2-T4 vs. Ta-T1	1.140	0.304	14.092	1	0.0002	3.126	1.724	5.668
Expression group, high vs. low	0.832	0.267	9.690	1	0.0018	2.297	1.361	3.879
Pathological tumor grade high vs. low	−0.951	0.410	5.386	1	0.0203	0.386	0.173	0.863
Recurrence status, yes vs. no	−1.420	0.647	4.814	1	0.0282	0.242	0.068	0.859

df, degree of freedom; HR, hazard ratio; CI, confidence interval.

## Data Availability

The datasets used and/or analyzed during the present study are available from the corresponding author upon reasonable request.
